# Performance of the Self‐Controlled Case Series for Drug Safety Signal Detection: A Multi‐Database Study

**DOI:** 10.1002/pds.70298

**Published:** 2026-02-12

**Authors:** Astrid Coste, Angel Y. S. Wong, Francois Haguinet, Andrew Bate, Ian J. Douglas

**Affiliations:** ^1^ Department of Non‐Communicable Diseases Epidemiology LSHTM London UK; ^2^ GSK London UK

**Keywords:** claims data, electronic health records, pharmacoepidemiology, pharmacovigilance, real‐world data, self‐controlled case series, signal detection

## Abstract

**Background:**

Differences in performance of the Self‐Controlled Case Series (SCCS) for signal detection have been reported across different databases. However, there has been limited comparative analysis of performance and it remains unknown whether combinations of databases could enable more effective signal detection.

**Objectives:**

This study aims to compare the performance of the SCCS for signal detection across several data sources, and to determine whether combinations of databases can improve SCCS performance.

**Methods:**

We applied the SCCS to macrolides and fluoroquinolone antibiotics, in four databases: Merative MarketScan Commercial Claims and Medicare, the Clinical Practice Research Datalink (CPRD) Aurum and the Système National des Données de Santé. We developed a reference set of 104 positive controls and 58 negative controls, using a taxonomy framework to ensure the selected drug outcome pairs are theoretically well suited to the SCCS design. The observation period lasted 2 years, with a 30‐day risk‐window after each dispensing. Diagnostic performance was measured using sensitivity, specificity and area under the receiver operating curve (AUC) with respect to the product labels, both for individual and combinations of databases.

**Results:**

The sensitivity of the SCCS ranged from 0.57–0.89 across individual databases, and the specificity from 0.43–0.77 when limited to drug‐outcome pairs sufficiently powered. The combination of all databases achieved the maximum sensitivity of 0.89 (0.41 specificity) for the full reference set, and a sensitivity of 1 (0.35 specificity) for drug outcome pairs with enough power. Whilst AUCs ranged from 0.66 to 0.71 across individual databases, the highest performing combination was CPRD plus MarketScan Commercial Claims (0.76 AUC).

**Conclusions:**

Using a carefully designed reference set of drug‐outcome pairs well suited to the study design, the SCCS performance varied substantially by database due to differences in population, reporting, healthcare and coding systems and prescribing patterns. Multi‐database studies showed increased performance of SCCS for signal detection.

## Key Points

- The Self‐Controlled Case Series (SCCS) is a promising method for drug safety signal detection using Real‐World Data but has not been implemented routinely because of heterogeneous performance reporting.
- We explored SCCS performance in individual and combinations of databases from three countries, using a tailored reference set based on macrolide and fluoroquinolone antibiotics drug labels.
- Sensitivity of and specificity of SCCS ranged from 0.57–0.89 and 0.43–0.77 respectively across individual databases when limited to drug‐outcome pairs sufficiently powered.
- Whilst AUCs ranged from 0.66 to 0.71 across individual databases, the highest performing combination was CPRD plus MarketScan Commercial Claims (0.76 AUC).
- Using a carefully designed reference set of drug‐outcome pairs well suited to the study design, SCCS performance varied substantially by database. Performance was increased when using multiple databases.

## Plain Language Summary

We explored the performance of a self‐controlled method, where risks of outcomes (potential adverse events) are compared between different time points in each patient, for the detection of Adverse Drug Reactions using health claims databases. We also explored the added value of a recent methodological development, active comparators, which can address some of the remaining bias. We looked at the capacity of the method at identifying known drug side effects for commonly prescribed antibiotics. The method was able to give a correct result in 57% to 89% of positive controls (known adverse drug events) depending on the databases, and 43% to 77% of negative controls (drugs known not to be associated with certain outcomes) when limited to drug‐outcome pairs with a satisfying number of occurrences. Overall, the performance of the method varied substantially by database, due to differences in population, healthcare systems and prescribing patterns in the different countries. Using combinations of databases enabled an increase in the performance of the method.

## 1 Introduction

With the increasing availability of Electronic Health Records (EHRs) and claims data, there is now a variety of different Real‐World Data (RWD) sources available for pharmacoepidemiologic research [1]. The potential for conducting multiple databases analyses, including for signal detection, is technically easier than ever before. One under‐researched question is which structured RWD source(s), or combination thereof, should be used for signal detection [2]. It is likely that different RWD sources will be differentially valuable for various types of exposures and outcomes, and possibly key covariates. However, using multiple databases might dilute signal detection capability, and overuse of RWD could also prevent the ability to keep RWD independent for formal hypothesis testing studies [3]. Therefore, we require evidence that use of RWD data for signal detection is likely to have a beneficial impact.

Multi‐database analyses are now common in pharmacoepidemiology, whether as one‐off studies or across established networks e.g., FDA Sentinel, DARWIN, ASPEN [4, 5, 6]. Some signal detection performance evaluation studies reported usage across multiple databases, but evidence of the usefulness of such an approach is required. Example questions include whether differential performance is seen across databases when applying a common reference set and whether performance increases using a combination of RWD sources.

Several projects have conducted multi‐country analyses for signal detection. Using both European and American data allows for triangulation of findings due to differences in health systems. Indeed, EHRs, commercial and administrative claims databases present differences in the data captured: primary care versus hospital diagnoses, clinical information, population coverage, coding systems, etc. [7].

Among published studies that investigated methods performances for signal detection in a multi‐database context, individual measures of performance for each data source were most often reported [8, 9, 10]. To our knowledge, no published work reported the differences in performance using different approaches to combine data sources.

The aim of this study is to compare the performance of the Self‐Controlled Case Series (SCCS), one of the more promising methods for signal detection [11], in four databases of different types: the Merative MarketScan Commercial Claims and Medicare databases, the Clinical Practice Research Datalink (CPRD) Aurum and the Systeme National des Donnees de Sante (SNDS). We will explore reasons for differential performance across databases and whether using a combination of databases could increase SCCS performance for signal detection. We used a specific reference set developed for this study using a taxonomy framework to ensure the selected drug outcome pairs are theoretically well suited to the SCCS design [12]. Using different types of data sources allows us to compare different database settings for signal detection [13].

## 2 Methods

### 2.1 Data Sources

We used four data sources from three countries: CPRD Aurum, Merative MarketScan Commercial Claims and Medicare, and the SNDS. The main characteristics of each data source are summarised in Table 1. We only used de‐identified patient‐level data; therefore, individual informed consent was not required.

**TABLE 1:** Description of the databases used in this study.

	CPRD Aurum	CCAE	Medicare	SNDS
Country	UK	US	US	France
Type	Electronic health records	Commercial claims	Commercial claims	Administrative claims
Population	Representative sample of the UK population 39 million patients (13 million active) as of 2021	Data from active employees and dependents, and early retirees (non‐Medicare) 38.7 million in 2019–2021	Data from active employees and retirees aged 65+, as well as dependents 2.3 million in 2019–2021	French inhabitants 68 million (> 99% of French population)
Information contained	De‐identified coded clinical records of symptoms, diagnoses, prescriptions, tests, demographic and lifestyle factors and referrals recorded in primary care [14]	De‐identified data from in‐ and out‐ patient visits and prescription claims Includes demographics, diagnoses, procedures, pharmacy claims and cost information.	De‐identified data from in‐ and out‐ patient visits and prescription claims Includes demographics, diagnoses, procedures, pharmacy claims and cost information.	De‐identified information on care reimbursement Outpatient data: patient information, drug dispensation, long term diseases, nature of laboratory tests Hospital diagnoses
Missing information and limitations	Hospital diagnoses (but linkages possible). Drugs delivered in hospital. Prescribed but not dispensed drugs.	Not representative of the US population. Poor recording of smoking status and BMI (linkage possible for a subset of the population).	Not representative of the US population. Poor recording of smoking status and BMI (linkage possible for a subset of the population).	BMI, smoking status, laboratory tests results, primary care diagnoses, drug dispensing in hospital.
Diagnosis coding scheme	SNOMED	ICD‐9CM ICD‐10	ICD‐9CM ICD‐10	ICD‐10
Use in signal detection studies	Used for near‐real time vaccine surveillance [15]. Not part of the structured initiatives for performance assessment such as EU‐ADR.	Used in the Observational Medical Outcomes Partnership (OMOP) initiative for performance assessment of methods for signal detection [8, 16].	Used in the OMOP initiative for performance assessment of methods for signal detection [8, 16].	Used in one project looking at the performance of SCCS for signal detection using two outcomes [17, 18].

Abbreviation: BMI, body mass index.

A Common Data Model (CDM) was not used in this study but instead, the same study protocol described below was applied to all data sources independently.

### 2.2 Study Design

The SCCS is a case‐only design comparing the event rate during exposed and unexposed time within the same individual [19]. Since all comparisons are made within person, time‐invariant, between‐person confounding is inherently addressed. Assumptions of this method can be found in the Supporting Information.

### 2.3 Reference Set

A reference set was designed specifically for this study using a taxonomy framework to ensure the selected drug outcome pairs are well suited to the SCCS design. The full process is detailed in a previous paper [12]. The reference set contains 104 positive controls (drug‐outcome pairs) and 58 negative controls in total, based on 30 outcomes from all organ classes.

Positive and negative control outcomes were selected among outcomes present on UK labels for macrolide and fluoroquinolone antibiotics, on an individual drug basis. Only clinically important outcomes meeting the SCCS requirements and a priori appropriately captured in all databases were considered in the reference set, based on a classification included in the taxonomy framework. UK labels present minor differences with the American and French versions.

### 2.4 Study Population and Design Choices

The observation period lasted 2 years from the 1 January 2017 to the 31 December 2018. The risk window lasted 30 days and began on the day after the dispensing/prescription date. We conducted a crude analysis assuming no relevant time varying confounders. Participants were eligible for inclusion at the latest of 1 year post registration or their 18th birthday. The population consisted of all adults with at least one oral macrolide (azithromycin, erythromycin, clarithromycin) or fluoroquinolone (FQ) (ciprofloxacin, ofloxacin, levofloxacin, moxifloxacin) prescription during the observation period. More details can be found in the Supporting Information.

### 2.5 Statistical Analyses and Measures of Performance

Performance was measured using sensitivity and specificity with respect to the UK product labels. Positive and negative predictive values have been reported in other works but were biased in this study due to an unequal number of positive and negative controls in the reference set. A common reason for inability to highlight safety signals is insufficient statistical power, as Adverse Drug Events (ADRs) are often rare. We therefore also explored performance of drug‐outcome pairs where there was apparent sufficient power. We considered satisfactory power for drug‐outcomes pairs with at least five events in the risk window, and where the upper bound of the 95% confidence interval (CI) Risk Ratio (RR) was < 2 for outcomes with no evidence of an association, e.g., RR = 1.55 (95% CI: 0.90–2.10) would not be considered satisfactory power.

For the purpose of this paper, the term “signals”, i.e., drug outcome pairs with a lower bound of the IRR 95% CI ≥ 1 will be used to denote Signal of Disproportionate Recording (SDR). This terminology is analogous to that used in spontaneous reporting systems to designate quantitative findings that would require clinical evaluation before they could be considered signals of suspected causality, where quantitative signal detection has been studied far more extensively. ‘Signals’ discussed herein have not undergone any clinical review.

### 2.6 Multi‐Database Analyses for Signal Detection

Several metrics were used to investigate the usefulness of a multi‐database study for signal detection. Firstly, we examined agreement between all databases to determine in what proportion of the reference set the different data sources led to the same results. This was calculated as the number of positive and negative controls which produced the same results in all databases (i.e., number of true positive in all databases, respectively true negative, false negative and false positive). 2 × 2 agreements were also computed.

Secondly, we assessed the value of database combinations in terms of SCCS performance. For this, we derived pooled sensitivities and specificities by determining the number of positive controls correctly picked up in at least one of the four databases, and repetitively for at least 2, 3 and all databases, both for the full reference set and for drug‐outcome pairs with sufficient power in all databases. This corresponds to eight sensitivities and specificities in total.

We also looked at the number of positive controls correctly picked up in at least one data source (‘at least one database’ setting thereafter) for all possible combinations of databases (e.g., positive controls picked up in CPRD or the SNDS). We repeated the same analysis for positive controls picked up in all databases (‘all databases’ setting thereafter) for all possible combinations (e.g., positive controls picked up in CPRD and SNDS). This represented 30 sensitivities and specificities in total, 15 for each setting. These metrics were computed both for all drug outcome pairs and for drug outcome pairs with sufficient power in all databases of the combination.

We also explored the highest performing combinations and settings in terms of area under the receiver operating characteristic curve (AUC).

## 3 Results

There were 144 574 patients included in CPRD, 988 095 in CCAE, 177 431 in Medicare and 703 562 in SNDS. Mean age ranged from 46.8 years old in CCAE to 76.5 in Medicare. Population characteristics in terms of age, sex, length of follow‐up, numbers of antibiotic prescriptions and deaths during the observation period varied by database (Table 2).

**TABLE 2:** Comparison of the different populations.

	CPRD	CCAE	Medicare	SNDS
Total population^a^	144 574	988 095	177 431	703 562
Age at cohort entry				
Mean (SD)	64.5 (18.5)	46.8 (12.6)	76.5 (8.0)	70.9 (16.4)
Median (IQR)	68 (27)	49 (20)	75 (12)	73 (22)
Sex (%)				
Female	60.8	64.1	50.3	47.6
Male	39.2	35.9	49.7	52.4
Mean length of follow‐Up (days)	688.7	639.0	609.7	668.0
Number of antibiotic prescriptions^b^				
Mean (SD)	4.1 (6.6)	2.8 (2.1)	3.2 (2.5)	2.7 (2.6)
Median (IQR)	2 (3)	2 (2)	2 (3)	2 (2)
Proportion of people dying during the observation period	7.8%	No death data recorded	No death data recorded	19.3%

^a^ To be included in the population, patients need to have at least one exposure to a drug of interest and one of the outcomes during the observation period.

^b^ The number of prescriptions/dispensing per patient of the seven drugs considered in this study during the observation period.

Table 3 shows the sensitivity and specificity of SCCS in all data sources for all drug‐outcome pairs and those with enough power, respectively. This ranged from 72 out of the 162 drug outcome pairs in CPRD to 119 in the SNDS.

**TABLE 3:** Measures of performance for SCCS in individual databases.

	All drug outcome pairs				Drug outcome pairs with enough power		
	Number of pairs	Number of pairs with no cases^a^	Sensitivity	Specificity	Number of pairs	Sensitivity	Specificity
CPRD	162	33	0.24	0.78	72	0.57	0.77
CCAE	162	25	0.55	0.76	116	0.73	0.68
Medicare	162	34	0.42	0.81	84	0.72	0.62
SNDS	162	6	0.68	0.60	119	0.89	0.43

^a^ In the baseline and/or risk period.

### 3.1 Agreement Between All Databases

The agreement across all the databases was 33.5% for the full reference set, and 46.0% when measured on the 51 drug‐outcome pairs which were powered enough in all four databases. 2 × 2 agreements for pairs with sufficient power can be found in the Supporting Information. The highest agreement was between CCAE and Medicare (84.5%) and the lowest between CPRD and CCAE (61.2%) and between CPRD and SNDS (61.2%).

### 3.2 Combinations of Databases

We computed overall measures of sensitivity and specificity (Table 4) for positive and negative controls correctly identified in respectively at least one, two, three or all databases. The maximum sensitivity was observed for positive controls highlighted as signals in at least one database, with 0.88 for the full reference set, and 1 when limited to drug‐outcome pairs with enough power. The corresponding specificities were 0.41 and 0.31. The maximum specificity was achieved for SDRs picked up in all databases for the full reference set, at 0.90, with a corresponding sensitivity of 0.13.

**TABLE 4:** Pooled measures of performance across all databases using the full reference set (162 pairs) and pairs with sufficient power in all databases (51 pairs).

Criteria true positive	SDR picked up in at least one of the four databases		SDR picked up in at least two of the four databases		SDR picked up in at least three of the four databases		SDR picked up in all databases	
	Full ref set	Sufficient power	Full ref set	Sufficient power	Full ref set	Sufficient power	Full ref set	Sufficient power
Sensitivity	0.88	1	0.57	0.89	0.30	0.71	0.13	0.38
Specificity	0.41	0.31	0.74	0.56	0.86	0.63	0.90	0.63

**FIGURE 1:**
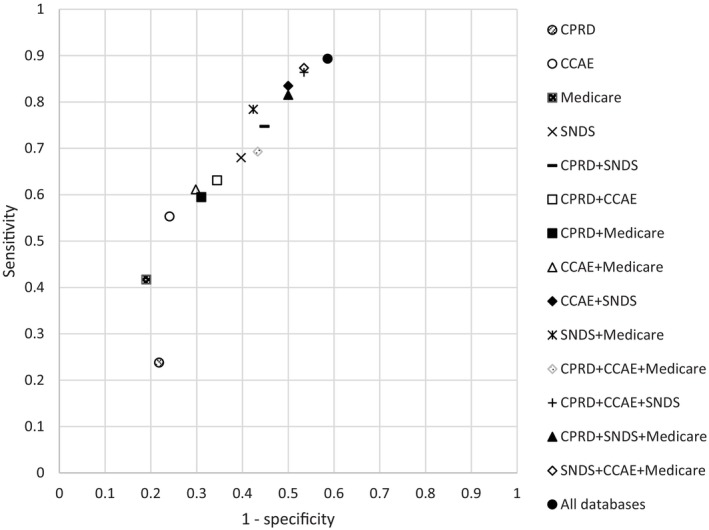
Performance of SCCS for individual databases and combinations of databases for the full reference set (162 pairs) ‘At least one database’ setting.

The sensitivity and specificity for all individual databases and combinations of 2, 3 and 4 databases using the ‘at least one database’ setting as described in the methods are plotted in Figure 1 for the full reference set. Plots for the ‘at least one database’ setting for pairs with sufficient power and for the ‘all databases’ setting can be found in the Supporting Information. The sensitivity ranged from 0.24 to 0.89 for the full reference set, and from 0.57 to 1 when limited to drug‐outcome pairs with sufficient power. The specificity ranged from 0.41 to 0.78 and from 0.25 to 0.68, respectively. In the ‘all databases’ setting, the sensitivity was in the range 0.12–0.39 for the full reference set, with specificities ranging from 0.82–0.91 depending on the combination of data sources. When limited to drug outcome pairs with enough power, the sensitivity was in the range 0.34–0.67 and the specificity ranged between 0.61–0.74. Plots for the ‘all databases’ setting can be found in the Supporting Information.

Whilst individual databases had AUCs of 0.66–0.71, the highest performing combination of data sources was CPRD plus CCAE, with an AUC of 0.76. The agreement between databases ranged from 61.2% between CPRD and CCAE and between SNDS and CPRD, and 84.5% between CCAE and Medicare (Table 5).

**TABLE 5:** Agreement between databases—Pairs with sufficient power only.

	CPRD	CCAE	Medicare	SNDS
CPRD	—	—	—	—
CCAE	61.2%	—	—	—
Medicare	62.3%	84.5%	—	—
SNDS	61.2%	69.6%	66.7%	—

## 4 Discussion

Our study using four different data sources across three countries showed that the SCCS performance varied substantially by database. When limited to drug‐outcome pairs with enough power, the SNDS was the highest performing database in terms of sensitivity (0.89) but also had the lowest specificity (0.43). CCAE and Medicare had very similar performances and achieved a good balance between sensitivity and specificity. As the database with the lowest power, CPRD achieved the lowest sensitivity but a high specificity.

Our results suggest that applying a multi‐database approach has advantages for signal detection in RWD, over the use of an individual database. Combinations of databases showed large increases in sensitivity compared with individual data sources for the full reference set, with a maximum of 0.89. Indeed, using multiple data sources should allow more SDRs to be highlighted for a broader range of outcomes, depending on their characteristics and population, although this needs to be confirmed in future studies. Several combinations of data sources also achieved high AUCs in this study. For example, the combination between CPRD and CCAE had an AUC of 0.76 for drug outcomes pairs with enough power and positive controls found in at least one of the two databases. Furthermore, an advantage of conducting a multi‐database study compared to a meta‐analysis of results from different studies is to reduce the heterogeneity by applying a common study protocol [20].

Our results for individual databases are consistent with previously published studies, notably from the OMOP and EU‐ADR projects. They reported average AUCs of 0.57–0.74 for SCCS in signal detection, across all databases and outcomes, although performance varied significantly by outcome and analytic setting [8, 9, 16, 21, 22]. Our study showed that using combinations of databases could achieve higher AUCs than the ones reported for individual databases in previous studies.

In signal detection, more emphasis is usually given to the sensitivity, due to the importance of identifying as many signals as possible, at the cost of introducing false positives. The AUC, which has been considered in previous studies as the primary measure of performance, may not be optimal since the same weight is given to the sensitivity and specificity. Among combinations of databases tested in this study, the ones leading to the highest sensitivity may be more informative for signal detection despite achieving lower AUCs than others. However, in our study, improvements in sensitivity when combining data sources led to a reduction in specificity. In a real world application this would mean having to triage and potentially investigate more signals. There is no consensus in the literature on minimum thresholds of sensitivity or specificity for signal detection. This should be discussed on a case by case basis as algorithms for RWD are only one component of an overall effective signal detection programme.

Most previous studies using SCCS for signal detection presented performance metrics after applying a minimum power threshold. Whilst the performance limited to drug‐outcome pairs meeting the power threshold is closer to the optimal performance of the method, the all pairs approach results in metrics closer to the real‐life performance and combines database weaknesses in terms of both size and potential bias. The power threshold in this study is arbitrary, and low sensitivity in CPRD might still be because of low numbers of cases compared with other data sources, despite the power threshold being met.

Eleven positive controls were missed in all four databases, and none of these pairs met the power threshold in any of the data sources. It is therefore not possible to determine whether these signals were missed due to insufficient power or other reasons. When restricting to drug outcome pairs with sufficient power in all databases, all positive controls were highlighted in at least one database (sensitivity of 1), but 4 of them (7.8%) were highlighted in one database only. For example, the positive control ciprofloxacin/tendon rupture was only highlighted in the SNDS. There is mixed evidence of an association in the literature, which might be because an increased risk is present only in certain subgroups (e.g., elderly patients with concomitant corticosteroids exposure) [23, 24, 25]. This example highlights that, whilst drug labels were used as a ‘gold standard’, they include ADRs with various levels of evidence.

Six negative controls were incorrectly highlighted as SDRs in all four databases, all related to pneumonia, cellulitis, and upper gastrointestinal bleeding. Confounding by indication may explain the observed elevated risk of these three outcomes. Using active comparators could mitigate confounding by indication and improve the specificity of the method for signal detection, although we have shown that the sensitivity is greatly decreased when active comparators are used in SCCS.

All four databases achieved the same result in 46% of drug outcome pairs with sufficient power. 2x2 agreements achieved high numbers nearing or above 70%, except CPRD, which led to more differences with other databases. These differences in the results might be explained by several factors: first, CPRD uses a different coding system for the outcomes (SNOMED) than other databases (ICD‐10), which led to differences in codelists. Databases considered in this study were of different types: CPRD Aurum only includes primary care data whilst other databases mainly include secondary care diagnoses. Therefore, the cases identified in CPRD might be less severe than those in other databases (Table 2). There were also differences in antibiotic prescribing between three countries [26, 27, 28], which might partly account for the differences in power across databases.

Because of these differences, database choice is a crucial element at study design stage. A guide for database selection in pharmacoepidemiology research has already been published [29]. Practically, the main reasons for choosing a database include the information contained e.g., EHRs vs. claims, primary vs. secondary care, cost, ease of access etc. In our work, we found that CPRD was not powered enough for a majority of the reference set, despite using commonly prescribed antibiotics. In this case, only common outcomes managed in primary care e.g., tendinitis had sufficient sample sizes. Another approach for multi‐database use involves conducting a meta‐analysis, which could increase statistical power for rare or poorly covered outcomes. More generally, using multiple databases for real‐life signal detection studies provides stronger evidence to detect signals representing true associations. Similarly, drug‐outcome pairs which did not stand out in several databases are less likely to be causally associated. The optimal combinations of databases are likely to depend on the nature of the drug and outcome under investigation, similarly to the considerations we applied on study design. This should be investigated in future work, as well as whether the entirety of a database should be used for signal detection, or whether a part should be preserved for future signal assessment studies. Moreover, SDRs detected in RWD, like in individual case safety reports, are leads that require further consideration from a clinical perspective. We accept that the nature of clinical investigation will be very different in RWD where for example clinical evaluation of individual records in claims databases may be unfeasible. Further research into how clinical and other considerations should be best done in the review of SDRs in RWD is needed. Signal detection is the first step in the signal management process. Once it has arisen, a signal needs to be prioritised and validated to determine whether there is sufficient evidence of a potential new causal association to justify further assessment. After passing this stage, the signal is evaluated using a combination of sources, including RWD, to determine if actions need to be taken. Finally, results of this study indicate that using the SCCS for signal detection does not work perfectly and generates some noise, even when using combinations of databases. However, this is also the case of quantitative algorithms used in spontaneous reporting systems [30].

### 4.1 Strengths and Limitations

The reference set included 30 outcomes of all organ classes, which is larger than previous studies investigating methods' performances in a similar way. Although it was limited to antibiotics, the results should generalise to any drug well suited to the SCCS requirements. All drugs and outcomes we selected were theoretically well suited to the SCCS design. This was done using a taxonomy framework incorporating SCCS assumptions, clinical importance of the outcomes and suitability to the database of interest among other criteria [12]. Such an approach has not yet been widely applied to signal detection and would benefit other studies to optimise performance.

Although we investigated the coverage of the drugs and outcomes of interest in the data sources at design stage, power remained a limitation for some drugs and outcomes in some databases. Therefore, relevant comparison across data sources was only possible on a subset of the initial reference set. In general, a challenge with the use of RWD for signal detection is that the data sources must have sufficient statistical power to effectively detect ADRs, particularly the rarer ones. Another limitation of this study was related to the selection of reference set based on labels. As outcomes are added to labels based on different levels of evidence, the presence of an outcome on the label does not necessarily imply a true causal association. Similarly, an outcome absent from a label could simply mean a causal association is not yet recognised. Labels also vary by country: UK labels presented minor differences with French and US labels for the drugs investigated in this study. Notably, it was not possible to have an ideal single reference set. Finally, using this reference set, we demonstrated the ability of SCCS to identify known ARDs and to exclude unrelated events. This performance may not translate in the real‐world detection of new signals.

## 5 Conclusions

We demonstrated that the performance of SCCS for signal detection varied significantly between four Real‐World databases, likely because of their differences in population, healthcare, coding systems, and prescribing patterns. Multi‐database studies present advantages for signal detection by providing more robust evidence on potential signals and should enable the prioritisation of certain drug outcome pairs for further evaluations studies. The optimal combinations of databases are likely to depend on the nature of the drug and outcome under investigation and should be discussed on a case‐by‐case basis.

## Funding

This work was supported by the GlaxoSmithKline.

## Ethics Statement

As we used de‐identified patient‐level data, individual informed consent was not required. The study was approved by the London School of Hygiene and Tropical Medicine Research Ethics Committee (Reference: 27650). The French study protocol was approved by the National Data Protection Agency, CNIL. The UK study protocol was approved by the Independent Scientific Advisory Committee for the Medicines and Healthcare products Regulatory Agency (No. 22_002362).

## Conflicts of Interest

A.C. is funded by a GSK PhD studentship to undertake this work. A.B. is an employee of GSK and holds stocks and stock options. F.H. is an employee of GSK and holds financial equities in GSK. I.J.D. holds grants and shares from GSK. GSK markets the following drugs: Augmentin. However A.B. and F.H. did not actively participate in the assessment of the labels and choice of outcomes for this methodological study.

## Supporting information


**Data S1:** Supporting Information.
