# Supplementary material for: Performance of the Self‐Controlled Case Series for Drug Safety Signal Detection: A Multi‐Database Study
Source: Pharmacoepidemiol Drug Saf. 2026 Feb 12;35(2):e70298. doi: 10.1002/pds.70298 (PMC12901761; doi:10.1002/pds.70298)
Supplement: Supplementary file 1 — Data S1: Supporting Information. [file PDS-35-e70298-s001.docx]

### SCCS assumptions

- Conditionally independent events [1]. In the main analysis, we only considered the first occurrence of an event to meet this assumption [2].
- An event does not influence subsequent exposures. A 30-day pre-exposure window was introduced to acknowledge that this assumption may be temporarily violated [2].
- An event does not influence the end of the observation period [3]. We ruled out outcomes leading to modest to large increases in mortality to meet this assumption in this study.
- Since accurate timing of exposure and outcome is crucial to SCCS [1], this method is better suited to transient exposures and acute outcomes. In this study, we considered antibiotics, which are usually prescribed in courses of less than 10 days. All chosen outcomes are acute, and we anticipate minimal misclassification of the time of occurrence.

### Figure – Performance of SCCS for individual databases and combinations of databases for drug outcome pairs with sufficient power – ‘At least one database’ setting

### Figure – Performance of SCCS for individual databases and combinations of databases for the full reference set – ‘All databases setting

### Figure - Performance of SCCS for individual databases and combinations of databases for drug outcome pairs with sufficient power – ‘All databases’ setting

### References

1. Zhou X, Douglas IanJ, Shen Rongjun, Bate Andrew, Douglas IanJ, Bate Andrew. Signal Detection for Recently Approved Products: Adapting and Evaluating Self-Controlled Case Series Method Using a US Claims and UK Electronic Medical Records Database. Drug Saf [Internet]. 2018 [cited 2021 Jul 13];41:523–36. Available from: http://rd.springer.com/journal/40264

2. Petersen I, Douglas I, Whitaker H. Self controlled case series methods: an alternative to standard epidemiological study designs. Br Med J [Internet]. 2016; Available from: http://dx.doi.org/10.1136/bmj.i4515

3. Whitaker HJ, Ghebremichael-Weldeselassie Y, Douglas IJ, Smeeth L, Farrington CP. Investigating the assumptions of the self-controlled case series method. Stat Med. 2018;37:643–58.
